# Visualizing the datasphere: Representations of old bodies and their data in promotional images of smart sensor technologies for aging at home

**DOI:** 10.3389/fsoc.2022.1008510

**Published:** 2022-12-20

**Authors:** Kirsten L. Ellison, Wendy Martin, Isabel Pedersen, Barbara L. Marshall

**Affiliations:** ^1^Department of Sociology, Trent University, Peterborough, ON, Canada; ^2^Department of Health Sciences, Brunel University London, Uxbridge, Middlesex, United Kingdom; ^3^Faculty of Social Sciences and Humanities, Ontario Tech University, Oshawa, ON, Canada

**Keywords:** socio-gerontechnology, aging-in-place, bodies, smart sensors, data visualization, data imaginary

## Abstract

Technologies for people aging at home are increasingly prevalent and include ambient monitoring devices that work together with wearables to remotely track and monitor older adults' biometric data and activities of daily living. There is, however, little research into the promotional and speculative images of technology-in-use. Our paper examines the ways in which the datafication of aging is offered up visually by technology companies to promote their products. Specifically, we ask: how are data visualized in promotional images of smart sensor technologies for aging at home? And in these visualizations, what happens to the aging body and relations of care? We include in our definition of smart sensor technologies both wearable and ambient monitoring devices, so long as they are used for the in-home passive monitoring of the inhabitant by a caregiver, excluding those devices targeted for institutional settings or those used for self-monitoring purposes. Our sample consists of 221 images collected between January and July of 2021 from the websites of 14 English-language companies that offer smart sensor technology for aging at home. Following a visual semiotic analysis, we present 3 themes on the visual representation of old bodies and their data: (1) Captured Data, (2) Spatialized Data, and (3) Networked Data. Each, we argue, contribute to a broader visualization of the “datasphere”. We conclude by highlighting the underlying assumptions of old bodies in the co-constitution of aging and technologies in which the fleshy and lived corporeality of bodies is more often lost, reduced to data points and automated care scenarios, and further disentangled from other bodies, contexts and things.

## Introduction

Alarmist discourses surrounding aging demographics and the increasing “care gap” due to aging populations are prevalent (Etkin, 2021; Orlov, 2022). It is in this context that new aging-in-place policies and technological innovations have been positioned as cost-effective and desirable solutions to the “problem of aging” (Peine and Neven, 2019). This is reflected in the Consumer Technology Association's projection that the U.S. market for technologies for aging at home would grow to nearly $30 billion by 2022 (Ewell, 2019). At the center of this projected growth were ambient monitoring devices that work together with wearables to remotely track and monitor older adults' biometric data and activities of daily living. Above all, these devices are promoted as offering caregivers constant access to information on the wellbeing of their loved ones, without needing to be present in the home, with the perceived objective to enhance peace of mind for the caregiver, and independence for the older adult.

The expansion of this market has been paired with a growing concern by science and technology studies and critical age studies scholars about the ways that privacy, control, surveillance and autonomy have been transformed and re-negotiated in the home with the introduction of these technologies and the implications this has for relations of care and power for older adults (Peek et al., 2015; Mortenson et al., 2016; Berridge and Wetle, 2019; Dalmer et al., 2022). Others have pointed to the symbolic, emotional and physical transformations of the home-space, emphasizing the very agentic nature of these devices and the data that they produce (Oudshoorn, 2012; Neven, 2015; Berridge, 2016). Of note, the assumed passivity of the users and the devices are questioned, and the co-constitutive relationship between aging and technology is highlighted. In this context, Peine and Neven (2021) have recently proposed an aging-technology co-constitutive model that involves four interconnected whilst distinct arenas: “Design Worlds,” “Technological Artifacts,” “Life Worlds of Older People,” and “Images of Aging,” each informing the other in a co-constitutive circuit. Highlighted in this model are the ways that the creative and technical worlds of innovation in the field of gerontechnology inform and are informed by both the lived and assumed realities of older people. “Images of Aging” form an integral arena in this model, existing as a bridge between the experiences and materialities of older people and the ways that they are imagined by the designers as future users of their devices. The images in this case refer to the way aging is imagined both by individuals and society, and perpetuated in mass media, fashion, and art (Peine and Neven, 2021, p. 2853). Absent from this model, however, are the promotional and speculative images of the devices themselves, functioning as a bridge between the technological artifacts and the ways that they are incorporated into the “Life Worlds of Older People”. As we argue, these images both define and reflect the kind of lifestyle that is imagined in the design of these technologies and inform the broader representational landscape of technologically-enabled “successful” aging. In the case of smart sensor technologies for aging at home, the promotional images work to sell a particular version of the digitally-enabled and data-driven lifestyle of the independent ager that is being sold alongside the technology itself.

Building on this, our paper examines the ways in which the datafication of aging is offered up visually by technology companies to promote their product. Specifically, we ask: how are data visualized in promotional images of smart sensor technologies for aging at home? And in these visualizations, what happens to the aging body and relations of care? Following a visual semiotic analysis of the images (Kress and van Leeuwen, 2021), our paper explores three themes in the visual representation of old bodies and their data within the home-space: (1) Captured Data, (2) Spatialized Data, and (3) Networked Data. Each, we argue, contribute to a broader visualization of the “datasphere”, a space that consists of the totality of the digital ecosystem, including the technical, physical, human, and political spaces through which data are produced, circulated and legitimized (Bergé, 2017; Douzet, 2020; Pedersen and Iliadis, 2020). In particular, we explore the underlying assumptions of old bodies in the co-constitution of aging and technologies in which the fleshy and lived corporeality of bodies is more often lost, reduced to data points and automated care scenarios, and further disentangled from other bodies, contexts and things. To conclude, our findings underscore tensions between enhancing independence whilst increasing surveillance of old bodies and the ways that space and place for older people at home are contested, complex and everchanging.

## Theoretical background

Our visual analysis of the datasphere and aging bodies is located on the broader terrain of critical data studies, which have explored, in different contexts, the processes through which human life is datafied (Lupton, 2016; Neff and Nafus, 2016; Ruckenstein and Schüll, 2017; Couldry and Mejias, 2019). The argument has been made that bodies are now viewed as data platforms that will “create new sources” for datafication, whereby “the labor of sensing, computing, energizing, storing (data), transmitting, and hosting a network” is further imposed on rapidly instrumentalized and commodified bodies (Pedersen, 2020, p. 24). Bodily movements and functions are rendered as data amenable to aggregation and analysis, providing a “means to *access, understand* and *monitor* peoples' behavior” (van Dijck, 2014, p. 198, emphasis in the original). Put bluntly, “the ‘body' here is… a data-generating device that must be coupled to sensor technology and analytic algorithms in order to be known” (Schüll, 2016, p. 326). As bodies are increasingly envisioned as components of data platforms, ambient technologies are justified as the means to extract data within enclosed spaces, incorporating “human subjects and vitalized bodies as mechanisms in larger dataspheres” (Pedersen, 2020, p. 27).

The potential for datafication to shape new forms of knowledge about bodies and lives has generated a variety of terms to capture the ways in which data from different sources are aggregated into profiles “through which selves become both objects and subjects of power in digital worlds” (Milne et al., 2022, p. 2–3). These include “data doubles” (Haggerty and Ericson, 2000; Ruckenstein, 2014), “data proxies” (Smith, 2016), “data selves” (Lupton, 2020) and “data shadows” (Leonelli et al., 2017). While all gesture toward ways of conceptually representing datafied body-subjects, acknowledging their partial and incomplete character, some, like “doubles” emphasize correspondence between data and body, while others, like “shadows,” seek to capture “the dynamic and distorted…nature of data representations” (Milne et al., 2022, p. 3). Where older bodies are concerned, these representations are less connected to data-subjects than data-objects—available for others to view, monitor and respond to, rather than shaping self and agency. The passivity of the data-emitting subject in this context suggests something akin to a “somatic portrait” assembled from the data and their analysis by others. As Milne et al. (2022) suggest, such “‘othered' data selves… pose potential threats to the life chances and choices of individuals, including when they are considered to be predictive of future problems” (p. 4).

Data analytics are, of course, inherently social—as Gitelman (2013) so aptly put it, “‘raw data' is an oxymoron”. To produce actionable knowledge—that is, to be comprehensible and have interpretive and predictive utility—they depend on culturally available narratives to furnish their stories with context and relevance (Dourish and Cruz, 2018). Importantly, datafication, as elaborated by Couldry and Mejias (2019), involves not just the measurement and quantification that transforms human life into data, but “the generation of different kinds of value from data” (p. 3). Beer (2019) coins the term “data imaginary” to describe the collated “promises and possibilities” offered up through the presentation of data analytics as: *speedy*, with data offered up in real-time; *accessible*, as analytics are delivered to mobile devices; *revealing*, capable of discerning unseen or unnoticed patterns of data; *panoramic*, providing a comprehensive view; *prophetic*, offering intimations of the future through predictive analytics; and *smart*, enabling better decision making (p. 21–35). Existing in the speculative realm of the datasphere, these promises and possibilities of data analytics are transformed *via* the data imaginary into solutions to problems, calling forth future-oriented idyllic visions of what could be.

When these promises and possibilities are tied to data technologies for aging bodies, they are dictated by a biomedical narrative of aging-as-decline, informing many key aspects of data collection, analysis and design (Neven and Peine, 2017; Cozza, 2021; Dalmer et al., 2022; Marshall et al., 2022). In contrast to the narrative of self-knowledge and corporeal optimization that informs, for example, members of the Quantified Self community, surveillance of older bodies is characterized by vigilant monitoring for signs of decline and management of risk (Neff and Nafus, 2016). These comprise what Monahan and Wall (2007) call “somatic surveillance systems,” in which the movements and measures of the bodies being surveilled become data, whose analysis offers up algorithmically determined decisions regarding their care. As they summarize it: “Body-monitoring systems translate corporeal information into data, relay this bodily data across information networks, and allow for the intervention of feedback mechanisms upon bodies” (2007, p. 162). Thus, when data imaginaries are enacted in the context of smart sensor technologies for aging at home, the speculative visions of data analytics are highlighted as persuasive, cost-efficient, and “smart” solutions to widespread concerns about how to care for an aging population (Neven and Peine, 2017; Marshall et al., 2022).

One of the ways in which the data imaginary is enacted and circulated is through the use of visual imagery (Beer, 2019, p. 35). Yet, to our knowledge, there have been no studies on the *visualization* of the data imaginary, with current research in the field of data visualization sidestepping the speculative realm of promotional imagery completely (Bihanic, 2015; Engebretsen and Kennedy, 2020). And while the stereotypical and dualist tendencies around alternative images of aging in the design and implementation of digital technologies for older people—such as active vs. passive, fit vs. frail—are well documented (Peine et al., 2021), there has been little consideration in the field of socio-gerontechnology of how the data imaginary of old bodies at home is represented visually. Moreover, despite an increase in visual methods in aging research (Martin, 2015), empirical explorations of the visual dimension of aging and the home-space in the context of smart technology have yet to include visual analyses of promotional imagery. This paper begins to fill these gaps, focusing specifically on what can be described as one of the most surveilled and controlled bodies in the datasphere: the old body. By examining the promotional images used to enact the data imaginary of smart sensor technologies for aging at home, our findings offer a novel visual exploration of the speculative realm of the datasphere, shedding light on a field worthy of inclusion in future explorations of the aging-technology co-constitutive circuit (Peine and Neven, 2021).

## Materials and methods

Between January and July of 2021^1^, our research team collected images found on the websites of 14 English-language companies that offer smart sensor technology for aging at home (Table 1). Screenshot captures were taken to preserve the original context and placement of the images.^2^ These companies were identified by conducting a Google search for aging-in-place technology and by scanning the exhibitor list of the 2021 Consumer Electronic Show. We include in our definition of smart sensor technologies both wearable and ambient monitoring devices, so long as they are used for the in-home passive monitoring of the inhabitant by a caregiver, excluding those devices intended for use in institutional settings or those used for *self*-monitoring purposes. Only images featured on pages with information for *home* technologies were collected, excluding those images used to promote institutional monitoring systems. Also excluded were companies whose products were either not on the market yet or did not yet have a webpage available. Our initial sample included 221 images. We then refined our image list to those that visualized data in some way. A total of 92 images were identified.

**Table 1:** Company/product name, website, access date, total images in initial sample, and total images visualizing data.

**Company/product (*n* = 14)**	**Website homepage**	**Date accessed**	**Total images (*n* = 221)**	**Data images (*n* = 92)**
Alcove	https://www.youralcove.com/	07/26/21	16	7
Best Buy Assured Living	https://www.bestbuy.ca/en-ca/services/best-buy-health-assured-living/	02/26/21	9	2
Billy	https://www.meetbilly.com	02/08/21	20	8
Caregiver Smart Solutions	https://www.caregiversmartsolutions.com/	02/26/21	7	1
CarePredict	https://www.carepredict.com/	01/19/21	20	8
EchoCare	http://www.echocare-tech.com/	02/25/21	13	9
Essence SmartCare	https://www.essencesmartcare.com	02/18/21	11	3
Forma SafeHome	https://www.formasafehome.com/	02/26/21	28	15
Howz	https://howz.com/	02/26/21	23	12
MySense	https://www.mysense.ai/#poweredByAIdesktop	02/18/21	26	5
Remote Home Check	https://www.remotehomecheck.com/	02/25/21	4	0
SofiHub Home	https://www.sofihub.com/	07/26/21	16	9
TrueSense	https://mytrusense.com/	02/24/21	16	6
Vayyar Home	https://vayyar.com/home/	07/26/21	12	7

**Figure 1:**
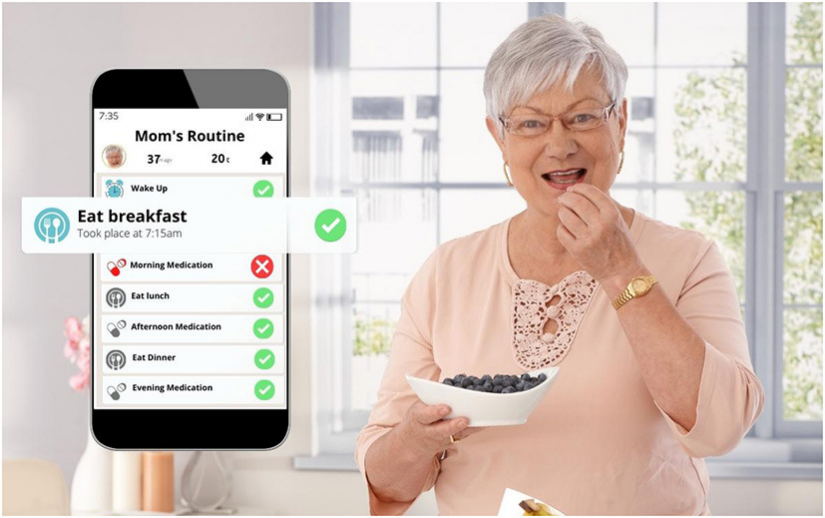
Mom's routine.

**Figure 2:**
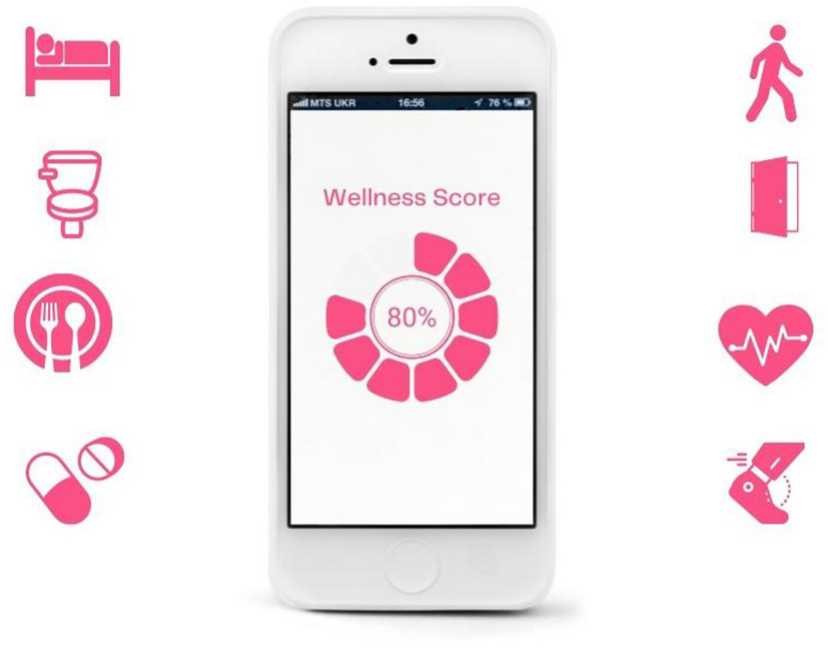
Daily score.

**Figure 3:**
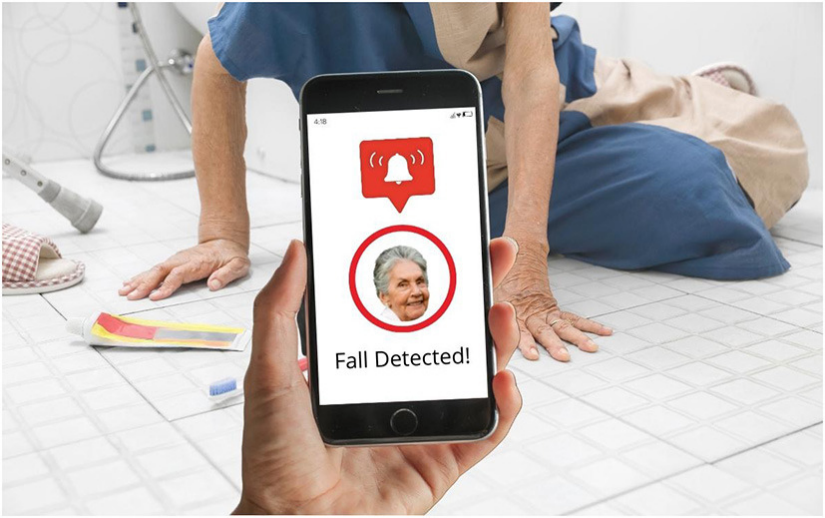
Fall detected.

**Figure 4:**
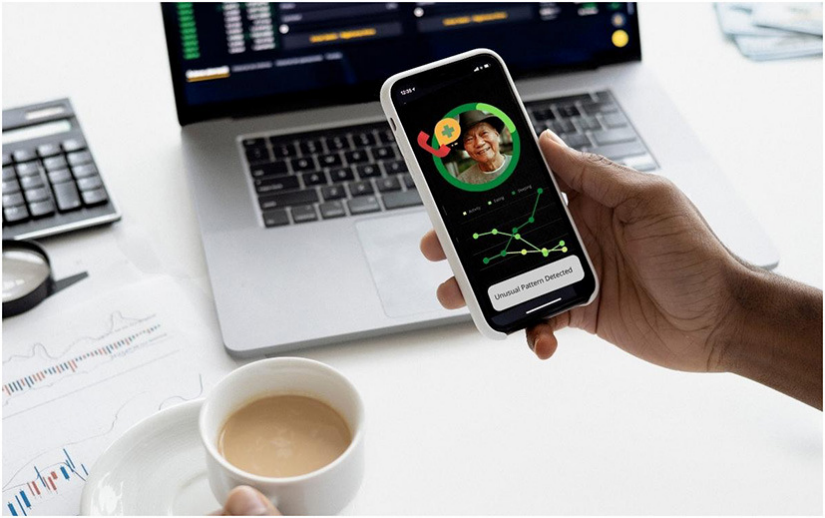
Progress report.

**Figure 5:**
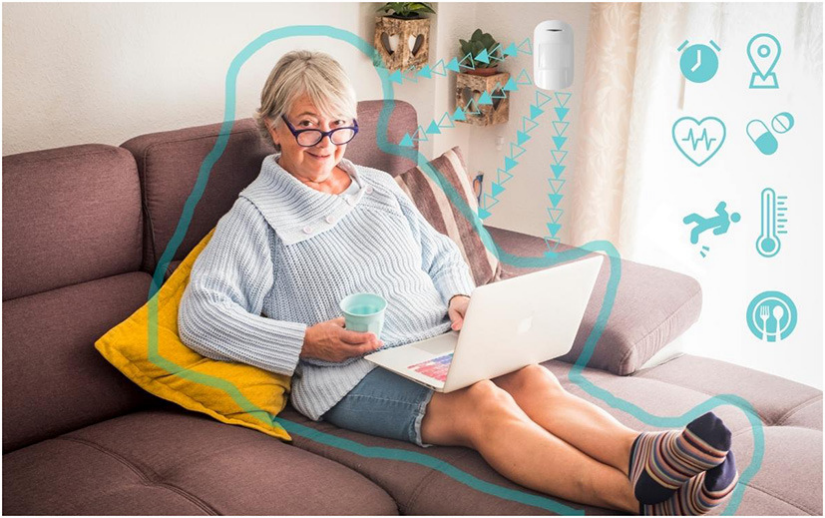
We've got you covered.

**Figure 6:**
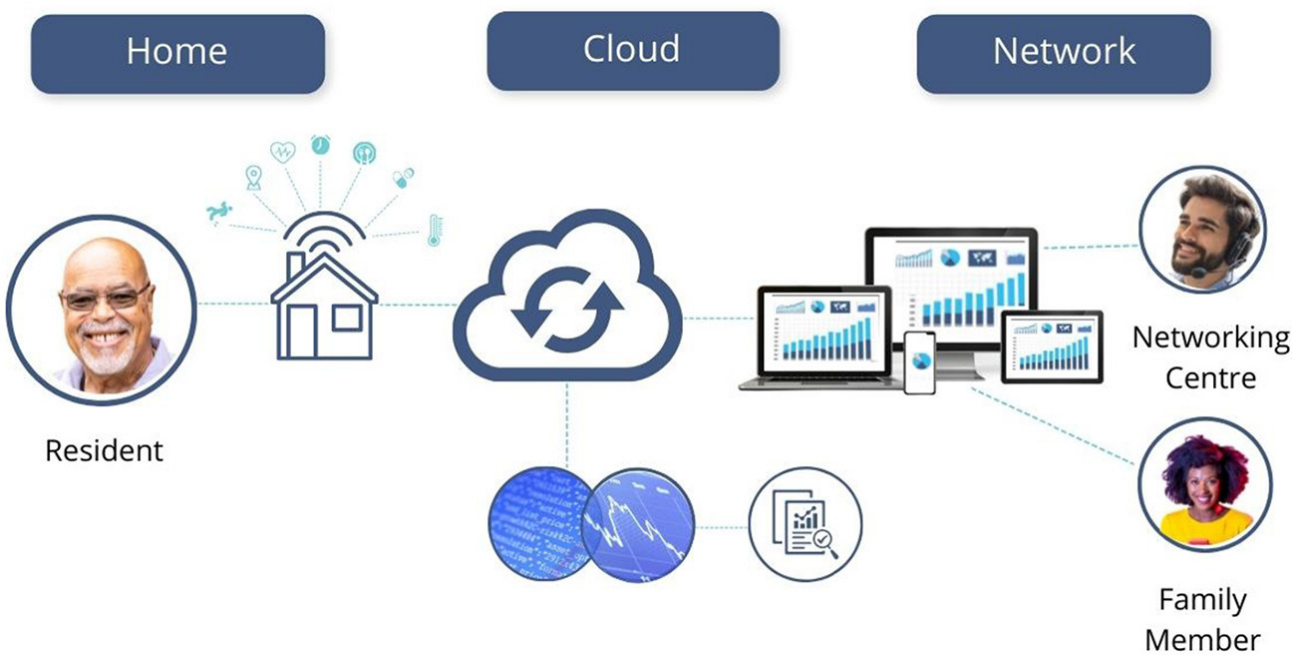
Network of care.

**Figure 7:**
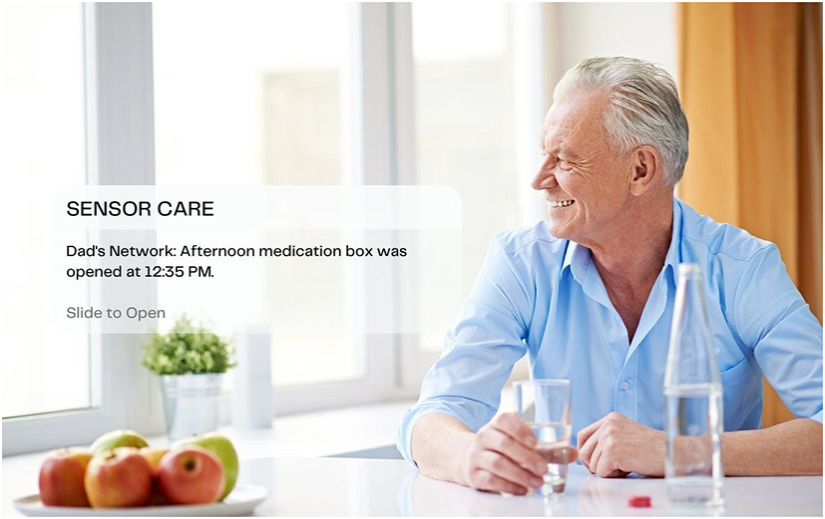
Dad's on track.

**Figure 8:**
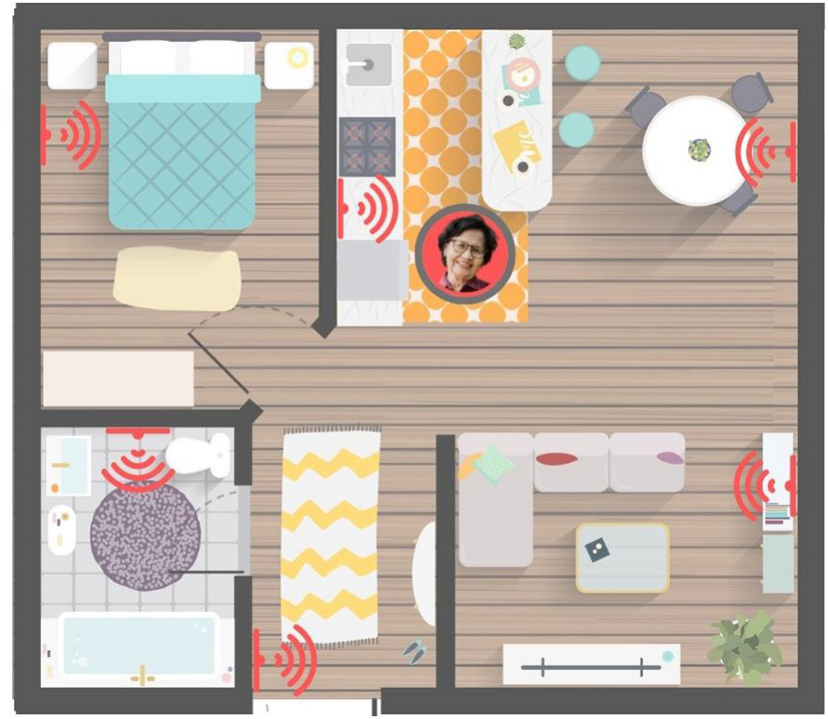
Safe and secure.

In her text on visual methodologies, Rose (2016) identifies three sites of image analysis: the production of the image, the content of the image, and the circulation of the image. Focusing our analysis on the still images captured from the company websites, we analyze the *content* of the image and its visual strategies of communication, asking *what* is depicted in the image and *how* (Rose, 2016; Russmann and Svensson, 2017). Drawing on the tenets of visual semiotics outlined by Kress and van Leeuwen (2021), we began with a close reading of the images to examine the visual strategies through which the datasphere is rendered visible, paying particular attention to the kinds of relationships to old bodies that they enable and promote. We use the term “strategies” to connote the techniques used to visually represent the data. Specifically, we examined the narrative, conceptual, modal, and compositional elements of the image, including the use of framing, positioning, perspective, and the presence or absence of represented participants, whether they be people or objects (Kress and van Leeuwen, 2021). We then grouped these strategies into three themes of data visualization: *Captured Data, Spatialized Data*, and *Networked Data*. In order to give the reader a sense of what these images looked like, the lead author created a series of visual mock-ups based on the most illustrative examples found in our sample (Figures 1–**8**). The images were created using the illustrator software, *Canva*, combining copyright-free stock imagery with graphics and text to recreate important visual elements and strategies that were identified in the sample.

## Results

### Captured Data

In the first theme, data are visualized and displayed within the frame of a mobile, handheld, or desktop device. Over half of the images in the sample (*n* = 50) include visual elements of Captured Data, totalling 64 data-capturing devices, including 50 mobile phones, 7 tablets, 6 computers, and 1 company-unique tabletop device. The prominence of this visual strategy is not surprising, given that one of the unifying features of these smart sensor technologies is that they are paired with a computer or smart phone application for caregivers to be able to access the older person's data remotely and with ease, and to be notified with daily updates and/or alerts. There are two components of this strategy that are of particular significance here: (1) the visualization of the device, and (2) the visualization of the data, captured within the device.

#### The visualization of the device

One of the key features of this strategy is that almost all of the data-capturing devices are depicted using either a frontal, objective perspective, or an angled, subjective perspective (Kress and van Leeuwen, 2021, p. 129, 140). In both cases, data are visually rendered actionable, demanding involvement on the part of the viewer. In the first case, the device is most often depicted floating either against a blank background or a natural setting, without a user or object holding it up (Figures 1, 2). The device is depicted at a 90-degree frontal angle, neutralizing any perspective of the image within it, reduced in these cases to a two-dimensional display. As Kress and van Leeuwen (2021) note, the lack of a central perspective encodes the image with an objective attitude toward the represented object(s), suggesting a privileged viewer position that reveals everything that is needed to be known about what is represented, a vantage point impossible in the real world (p. 140). The frontal angle, they argue, is an angle of *maximum involvement*, oriented toward action and displaying to the viewer “how it works” and “what to do” (p. 140). In other images, this same angle is used; however, the device is depicted in the notably *young* hand of the represented user, from a first-person perspective, with no visibility of the user's body (Figure 3). In these cases, the viewer is given a represented *youthful* form, drawn in as the central participant in action. The action-oriented objective display of the data is literally put into the young hands of the viewer invoking a sense of ownership and control over the device as tool and the data as objects to be acted on. The finger grazing over the display functions in these images as the vector of action, emanating from the viewer as represented user (Kress and van Leeuwen, 2021, p. 55–57).

In the remaining images, the device and its captured data are slightly angled, either floating in space, propped up on an object in a natural setting, or depicted in the bodyless young hand of the user (Figure 4). The use of an angle in these cases does not change the representational meaning of the data, but rather the viewer's level of involvement in it (Kress and van Leeuwen, 2021, p. 135). In images where the devices are depicted in the hand of the represented user, in a forward-leaning tilt, the oblique lines of the device create a sense of vectorality, positioning the viewer as engaged in a process of dynamic action with the device and the data (Kress and van Leeuwen, 2021, p. 55) (Figure 4). When the angled devices are depicted as *detached* from the represented user, either floating in space, or propped up on an object, the viewer is positioned as slightly less involved in the data captured in the device, which are offered up instead as a *possible* tool, available for action, yet not yet fully engaged.

#### The visualization of the data

Captured within the frame of these devices is a display of data that is intended to represent the somatic profile of the older person under surveillance. Although a handful of the depicted devices display application home screens, communication functions or back-end settings, most showcase the data as digital and quantitative composites of the older person in the form of lists, graphs, alerts, and scores (Figures 1–4). These images feature representational structures that are analytical in function, relating the whole (the older person) to its parts (the data), where the older person is only visible, knowable, and thus manageable, in the form of his or her data composite (Kress and van Leeuwen, 2021, p. 83). By its very nature, the visualization of data in the form of graphs, scores, and itemized lists of activities and bio-measurements invites a form of detached, impersonal scrutiny on the part of the viewer as represented user (Kress and van Leeuwen, 2021, p. 77). In some cases where the profile image of the older person is included in the frame of the device display, this image is backgrounded in a circular frame, standing in as synecdoche for the whole body, yet only given meaning and substance in the composition of data points that surround it (Figures 1, 3, 4). While itemized lists present daily scores and updates, graphs imply *movement*, offering a narrative snapshot of the older person's shifting somatic portrait through time (Kress and van Leeuwen, 2021, p. 94) (Figure 4). In both cases, whether in the moment or through time, the emphasis is on technical precision, accuracy, and quantitative purity, a clean extraction of individualizable and analyzable data points. When these measurements and activities are brought together to form a daily “score”, a holistic, self-contained image of the data is presented (often depicted within a circular frame), compounding the various data points together into a single image, with no space for extraneous data or context (Kress and van Leeuwen, 2021, p. 93) (Figure 2).

Aside from a handful of profile images of old faces, old *bodies* and their actual activities are backgrounded or completely absent in the composition of the images as a whole; all that is foregrounded is the data. In the three images where old bodies are present, they are backgrounded to the side of the image or in the far-off distance, or faded behind and obscured by the device, which is being held over the body-as-scene (Figures 1, 3). Functioning as *locative circumstances*, old bodies are reduced to nothing more than the contextual setting for the young hands of the viewer as represented user to act on the data being presented on the screen (Kress and van Leeuwen, 2006, p. 71). In the images where old bodies are completely absent from the background, they are replaced instead by unpopulated home, work, and outdoor settings, or blank space (Figures 2, 4).

### Spatialized Data

In the second strategy of data visualization, floating images of alerts, assessments, vectors, connectors, pulses, nodal points, and icons function to visualize the immaterial presence of data, giving it a spatiality it does not normally exhibit. Unlike the visualization of data in the form of graphs and scores visible on the screen of a device, this strategy of visualization gives data an imagined spatial presence *outside* of the device. A total of 46 images include visual elements of this strategy, which have been subdivided into three categories (1) data overlays, (2) data movement, and (3) data capture, each representing different ways that data are given a spatial presence.

#### Data overlays

In the first category of Spatialized Data, data is given a spatial presence in the form of an icon, text bubble, or screen extract that is depicted as floating in mid-space, either peripherally connected to the device from which they emanate or detached completely (Figures 1, 2, 5, 6, 7). The icons visualize the different categories of knowledge (and control) available *via* the sensor technology and its connected device. Visualized categories include sleeping, bathing, eating, cooking, toileting, taking medications, entering/exiting the home, fall detection, movement/activity tracking, emergency buttons, home security, and temperature control (Figures 2, 6). Floating text bubbles and screen extracts, on the other hand, take one of four forms: alerts to notify the caregiver of deviances from the older adults routines (missed medication, late wake-ups or bedtimes, missed meals); reports on the older adults' status (arrival at the home, activity levels, completion of tasks) (Figures 1, 7); communication with the older adult (reminders of doctor's appointments, suggestions to drink water, messages to and from their caregiver); and back-end controls to manage the tracking capabilities of the sensor technology. Of the 42 images that spatialize data, 34 include at least one of these visual elements.

When these images are against a white backdrop, the icons and text bubbles highlight the possibilities that these technologies possess, not contained within the screen of a device, but extracted out as tangible entities that have the power to monitor and connect with (from a distance) the absent bodies that they track (Figure 2). In the images where a background scene *is* present, it is through the lens of the icons or text bubbles that we are invited to “read” the scene. When these images include icon overlays, the home is transformed into a safe space, where its boundaries, environments, and inhabitants are secure, controllable, and analyzable from a distance, with the touch of an icon (Figure 5). Alternatively, in images of the home-space with floating reminders and messages for the (absent) older adult, the sensor technology and its networked data are given a material presence in the home, filling the empty interior with messages of care and companionship. Through the lens of floating icons, the home becomes a space of safety; through the lens of reminders and messages it becomes a space of comfort and connection.

When old bodies *are* depicted, they are reduced to the object of surveillance and/or intervention, whose activities and statuses become the lens through which their risk levels are assessed. Icons, alerts, and reports floating overtop of their bodies and surroundings reduce their corporeality into an analyzable and modifiable surface, a fleshy interface rendered clean and legible. Young bodies, conversely, are depicted once again as the users of the devices and the recipients of the data. Icons, alerts, and reports are peripherally connected with in-hand devices, extending their gaze and reach to spaces and bodies beyond the frame of the image (Figure 2). Through this lens, the caregiver as depicted user is granted unmediated and remote access to the old-body-as-data.

#### Data movement

In the second category, data are spatialized in the visualization of their movement across the home-space. What is imperceptible to the human eye is given visual form: vectored lines shooting across space, haloed edges, and curved signal pulses expanding the tangible horizon of the device's reach (Figures 5, 6, 8). Data are given not only a spatiality, but a *vitality*, moving and pulsing through space and time, reading and responding to all that falls within the periphery of their sprawling reach. And while many of these companies emphasize the unobtrusiveness of their sensors and wearables, these images visualize the very real material implications of these devices, transforming the home into a disciplinary space that not only watches and tracks the older person, but one that reduces everyday movements and activities into spaces of risk and potential intervention.

#### Data capture

In the final category of Spatialized Data, data's spatial presence captures and consumes the body-space of the older person. Unlike data overlays, this form of spatialization renders the *entirety* of the body-space into readable data. There are two visual elements that characterize this form of spatialization. The first is the visual wrapping of the older body in vectorial lines (Figure 5), like a lasso circling around the neck of its conquest. The body's corporeality has no escape from the gaze of the data sensors, enveloping it in a blanket of surveillance and analysis. The second replaces the corporeal body with its data composite, either as an assemblage of nodes in the shape of its figure, or as a singular bodyless picture or graphic icon (Figure 8). Standing as a synecdoche for the whole, the data composite completely erases the visibility of the older body as anything but data, present only in the form of information to be captured, read, and analyzed.

### Networked Data

In the final visual strategy, devices, data, bodies, faces, homes and other built structures are visually displayed within interconnected networks to signify relations of care. Unlike the other strategies that concentrate on spaces *inside* homes, Networked Data visualizes broader relations with actors outside the home. One dominant visual element of data as networked is the graphical style of wireless cloud computing. Some images depict illustrated floating “data clouds” (and in some cases the word “cloud”), linked to illustrated or photo realistic people through delicate monochrome lines (Figure 6). In this type of composition, faces are mapped with devices, indoor and outdoor places, and representations of activities as if operating in a working dynamic data system. The effect is that logical relations appear to occur in sequence between participants (Kress and van Leeuwen, 2021, p. 97). Assemblages of human agents, devices and servers are depicted within data clouds that can operate seamlessly. These images conceptualize relations with third-party organizations (tech support, practitioners, emergency workers), users (caregivers), and older people joined as if part of a single network. They imply a systemic working means of collecting disparate types of data from a variety of sources brought into neat ecosystems.

Another visual element of Networked Data is the representation of a symbolic center. An older face, an icon of a home, or even a product icon signifies the central hub for the network, which in turn appears to be the controlling entity for activities or relations across the network. Kress and van Leeuwen (2021) label these relationships as signifying a “central node,” with “radiating lines” connecting the “possessive attributes” (p. 100). These networks at times reveal an (in)visibility paradox within the images where old bodies and relations of care and caring are implied rather than visualized. Invisible are the labor, time, and movements needed to keep the network working; visible only are the mapped relations in a functioning, conceptual, topological network. Old bodies appear to integrate with seamless data flows; seamless interaction “has developed into a value-based governing logic” for biodata platforms that “should involve efficient, continuous interactions” (Pedersen, 2020, p. 29–30). Ultimately, independence and achieving independence is met by participating in the (successful) seamless network or serving as its central node.

## Discussion

A central goal of this paper is to build on the co-constitutive model of aging and technology proposed by Peine and Neven (2021) by illuminating an additional site warranting further exploration in the study of aging-technology relations: the *speculative* dimension of images of aging—in this case, the images of the data imaginary used to promote the promises and possibilities of smart sensor technologies for aging at home. Not only do these images feed back into the ways aging is imagined and the stereotypes that underlie these social imaginaries, but they are also informed by and reflective of the “age scripts” and assumptions built into the designs of the technologies being promoted (Neven, 2010; Bischof and Jarke, 2021; Joyce, 2021). Our visual analysis of these images offers novel insights into the ways old bodies are visualized within the datasphere, additionally contributing to the field of critical data studies and data visualization. Alongside visions of independent agers and liberated caregivers, these images offer visual narratives of the promises and possibilities of smart sensor technologies for aging at home where, (1) aging bodies are only visible as data to be tracked and managed, (2) the home-space is reconfigured into a disciplinary space of control and management, and (3) embodied care-work is disembodied and displaced from the home. Each of these are outlined below.

Firstly, our findings highlight an (in)visibility paradox in the visual representation of old bodies and their data. In the visualization of the datasphere, old bodies are both absent and present. On the one hand, the visibility of the old body is central. Smart sensor devices that are monitored by others are by their nature monitoring and observing the most intimate aspects of the body in everyday life, that includes movements, eating, and sleeping: in this context the old body is laid bare to be tracked, monitored, and rendered both visible and actionable *via* the remote screens of the caregiver's device. On the other hand, the corporeality of old bodies is either absent, backgrounded, and/or reduced to a disembodied somatic portrait, relocated within a data network of remote care. In other words, the old body's visibility is limited to the data produced by the ambient sensors and wearable devices that are aggregated and itemized into risk assessments and patterns of behavior.

The paradox of the absence/presence and (in)visibility of old bodies is well documented in aging studies (Gilleard and Higgs, 2018; Pilcher and Martin, 2020). In part, this is due to the ageist and gendered assumptions about old bodies in which visual representations of old age are created in a “visual culture that systematically devalues and erases age” (Twigg, 2013, p. 101) and in which old bodies are viewed as a disruption to the visual field (Hepworth, 2000). Gilleard and Higgs (2018) further distinguish between embodiment—the cultural and social dimensions of the body and practices that express identity and lifestyles of aging most often associated with the third age—and corporeality—the bodily and fleshy signs of aging through the lifecourse most often expressed as part of the imaginary of the fourth age. Corporeality, they argue, “represents the body as something that is reacted to—the objectivity of the person rather than his or her subjectivity” (2018, p. 9). In the context of our visual analysis, it is the corporeality of aging that is captured by data, representing the old body as something to be reacted to. At the same time, it is a corporeality that is both disembodied and datafied, purging it of its fleshy and lived entanglements. Thus, while the visibility of old bodies is central in the data imaginary of smart sensor technologies for aging at home, this visibility is partial, value-laden, and furthermore framed within the interventionist logic of risk management and cost efficiency (Peine and Neven, 2019).

Explorations into the representation of aging and old bodies in the context of ambient and wearable devices have drawn attention to the ways that dominant binaries of aging—active/passive, independent/dependent, fit/frail—are translated into quantifiable distinctions, offering measurable insights into a person's location on the scale from healthy, successful aging to unhealthy, vulnerable aging (Oxlund and Whyte, 2014; Marshall and Katz, 2016; Katz and Marshall, 2018; Dalmer et al., 2022). While many of the images in our initial sample included positive vs. negative images very similar to those identified in other studies on images of aging that fall along these aging binaries (Martin, 2012; Low and Dupuis-Blanchard, 2013; Hurd Clarke et al., 2014; Marshall and Rahman, 2015), our focus on the visualization of *data* sheds light on an expression of the active/passive binary that is unique to this topic. While there was a clear distinction between the fit vs. frail old bodies depicted in the background of the devices and their data, what is more compelling is the contrast between young hands and old bodies. In these images, whether the old body is fit or frail, active or passive, it is nonetheless always needing to be monitored *by someone else*, a distinctively *younger* someone else. The consistent use of young hands holding a device with access to and control over the data further cements this visibility of the older person as object of surveillance rather than subject of control. In this speculative vision, control lies in the hands of the end user of the data, with old bodies reduced to nothing more than data-emitting surfaces available to be “read” at any time.

Our findings furthermore offer novel insight into the reconfiguration of the home-space *via* ambient and remote care technologies. Recent research examining this topic has highlighted the lived experiences of older adults and their engagements with these technologies in the home (Oudshoorn, 2012; Mortenson et al., 2015, 2016; Neven, 2015; Berridge, 2016, 2017; Pol et al., 2016). As these scholars note, the home-space furnished by these tracking technologies is one where the symbolic divide between public and private life is eroded, and where expectations of intimacy, autonomy, and control are renegotiated in the name of safety and independence (Dalmer et al., 2022). As Roberts et al. (2019) have put it, the home in these cases is reconfigured into “a preclinical space, a kind of waiting room serviced by sensors and systems of monitoring” (p. 125). What our analysis adds to this research is an entry-point into the ways in which this reconfiguration of space is given visual form. As we argue, the spatialization of data—*via* data overlays, data movement, or data capture—not only grants the circulation of data a visibility, but also a vitality, and one that is *disciplinary* in nature: its primary function is to assess and report any deviations from predicted patterns of behavior. Overlayed messages—whether they be alerts, assessments, reminders, or communication between family members—additionally fill the space with messages of care. In the absence of caring *people*, floating and moving data offer a *presence* that fills the spaces between bodies, sensors, and remote devices. What is important to note is that these are all imagined visibilities; we cannot see data being received or transmitted, nor can we see (without the aid of augmented reality) messages or icons floating in mid-air. What these speculative visions offer is a visual narrative that reconfigures both the home-space and the body-space into the ever-expanding domains of the datasphere.

Finally, just as data are given a visual, spatial form, the labor of care is both displaced and disembodied. Absent from the clean lines connecting nodes, clouds, data, telecare operators and caregivers is the labor involved in caring for a loved one *via* these promoted systems of remote monitoring and data management. As Dalmer et al. (2022) note, “managing data care (updating, collecting, recording, interpreting, and relaying) becomes an added, but invisible burden to the already difficult and undervalued work of providing body care to older care recipients” (p. 88). In these images, the labor of care is invisible, replaced instead by vectors, connective lines, icons, and nodes that render the materiality and corporeality of data, devices, bodies, and relations of care into a seamless flow of information.

### Limitations and future directions: Between images, bodies, and technologies

Offering visions of technologically-enabled aging *futures*, the promotional images that exist between “Design Worlds” and the “Life Worlds of Older People” (Peine and Neven, 2021) must nonetheless be negotiated within the material contexts of technology-in-use, and the messy dynamics of data care, embodiment, and power (Dalmer et al., 2022). This paper is a starting point in identifying the dominant strategies through which old bodies and their data are given visual form. It is in the negotiation of these images, both in their creation and reception, that more work can be done. For example, our findings would be complemented greatly by ethnographic insight into the dialogue that occurs between designers, company executives, and marketing creatives to produce a vision that represents the kind of future promised by their product. Additionally, a greater understanding of the ways that intended users of these devices—older people and their family caregivers—interpret and respond to these images and the devices themselves would allow us to dig deeper into the dynamics at play in the co-constitution of aging and technology. Our analysis of the images is also limited to a specific analytic lens, focusing solely on the visualization of data and bracketing out the many other elements that contribute to the representation of older adults in the public domain. As a novel entry-point into the study of the visual dimension of the datasphere, our findings highlight the need for more researchers in critical data studies to move beyond data visualization, to images of the data imaginary. A fruitful starting point for this work would be to examine whether the identified strategies of Captured Data, Spatialized Data, and Networked Data resonate in data images existing outside the niche market of smart sensor technologies for aging at home.

## Data availability statement

The raw data supporting the conclusions of this article will be made available by the authors, without undue reservation.

## Author contributions

All authors listed have made a substantial, direct, and intellectual contribution to the work and approved it for publication.

## Footnotes

^1^As website pages are frequently updated and images can change over time, it is important to note that the images collected between February and July of 2021 may not all still be active and may no longer be representative of the images these companies currently have on their sites. Additionally, during the time of our image collection, the websites of Vayyar Home, Alcove, and SofiHub underwent significant changes. Thus, the images included in our sample are those that were available on their websites as of July 26, 2021.

^2^The images collected have been stored in a password-protected shared drive only accessible to the researchers and only used for research purposes. The images have not been reproduced or distributed.
